# The Effects of Physical and Mind–Body Exercise on Anxiety Among Patients with Breast Cancer: A Systematic Review and Meta-Analysis of Controlled Trials

**DOI:** 10.3390/cancers18193244

**Published:** 2026-10-08

**Authors:** Mesut Süleymanoğulları, Cemre Didem Eyipınar, Zarife Pancar, Valentina Stefanica, Nicola Luigi Bragazzi, Halil İbrahim Ceylan

**Affiliations:** 1 Division of Physical Education and Sport, Department of Physical Education and Sport, Faculty of Sport Sciences, Ağrı Ibrahim Çeçen University, 04100 Ağrı, Türkiye; msuleymanogullari@agri.edu.tr; 2 Department of Physical Education and Sport, Faculty of Sport Sciences, Gaziantep University, 27310 Gaziantep, Türkiye; cemreeyipinar@gantep.edu.tr (C.D.E.); z_pancar@hotmail.com (Z.P.); 3 Department of Physical Education and Sport, Faculty of Sciences, Physical Education and Informatics, National University of Science and Technology Politehnica Bucharest, Pitesti University Center, 110040 Pitesti, Romania; 4 Department of Clinical Pharmacy, Saarland University, 66123 Saarbrücken, Germany; nicola.bragazzi@uni-saarland.de; 5 Department of Physical Education of Sports Teaching, Faculty of Sports Sciences, Atatürk University, 25240 Erzurum, Türkiye

**Keywords:** breast neoplasms, mind–body therapies, exercise, anxiety, meta-analysis

## Abstract

**Background and Objectives:** Anxiety is highly prevalent among breast cancer patients and significantly impairs quality of life. Although both conventional physical exercise (PE) and mind–body interventions (MBE) are used in supportive care, their relative efficacy remains unclear. This systematic review and meta-analysis compared the effects of PE and MBE on anxiety in breast cancer patients. **Materials and Methods:** PubMed, Web of Science, Embase, PsycINFO, and CINAHL were systematically searched through April 2026 for controlled trials comparing structured PE or MBE with control conditions. Effect sizes were pooled as standardized mean differences (SMDs; Hedges’ g) using a random-effects model with restricted maximum likelihood estimation and modified Hartung–Knapp inference. Subgroup analyses and univariable meta-regressions explored potential moderators. **Results:** Twenty-four reports (*N* = 2197) represented 25 independent trials; 22 estimates from 21 reports entered the meta-analysis, and three reports were synthesized narratively. Interventions significantly reduced anxiety on average (SMD = −0.58, 95% CI [−1.01, −0.15], *p* = 0.011), with substantial heterogeneity (I^2^ = 85.0%) and a 95% prediction interval of −2.38 to 1.22. Both PE (SMD = −0.67) and MBE (SMD = −0.46) favored intervention numerically, although both subgroup confidence intervals included no effect, with no significant difference between modalities (*p* = 0.697). A larger, yet non-significant, effect was observed during active treatment (SMD = −0.84, 95% CI [−1.68, 0.01]) compared with survivorship (SMD = −0.34, 95% CI [−0.62, −0.07]); the between-phase comparison was inconclusive (*p* = 0.291). The comparison across diagnostic stages was also inconclusive (*p* = 0.366). None of the seven continuous moderators met the multiplicity-adjusted significance threshold. **Conclusions:** Current findings suggest an average reduction in anxiety following physical and mind–body interventions in breast cancer patients. However, no statistically significant differences were observed between intervention modalities or treatment stages. Very low certainty, substantial heterogeneity and indirect subgroup comparisons limit confidence in the size and consistency of benefit. Overall, individualized interventions may have a role in oncology care, but these findings do not establish superiority, equivalence or differential efficacy between modalities or treatment phases.

## Simple Summary

Anxiety is common among women with breast cancer and can negatively affect quality of life during and after treatment. Exercise is increasingly recommended as supportive care, but it remains unclear whether conventional physical exercise or mind–body interventions are more effective for reducing anxiety. This systematic review and meta-analysis compared these two approaches and examined whether their effects differed according to treatment phase and cancer stage. The findings showed an average reduction in anxiety across the pooled evidence, with no clear evidence that one approach was superior to the other. However, effects varied considerably between studies, and confidence in the evidence was very low. Comparisons across different phases of the cancer continuum and diagnostic stages were inconclusive. These results support further evaluation of individualized exercise programs in breast cancer care while highlighting the need for further research to determine which exercise characteristics may provide the greatest benefit for specific patient groups.

## 1. Introduction

Breast cancer remains the most frequently diagnosed malignancy and a leading cause of cancer-related mortality among women worldwide, with incidence rates continuing to rise and posing a substantial global health burden [1,2,3]. In 2022 alone, approximately 2.3 million new cases were reported, and this number is projected to exceed 3 million by 2040. Although advances in early detection and multimodal oncological treatments—including surgery, chemotherapy, radiotherapy, and endocrine therapy—have substantially improved survival rates, with 5-year relative survival exceeding 90% in many high-income countries with established screening and treatment infrastructure [4], considerable disparities persist, and survival rates remain markedly lower in many low- and middle-income countries due to later-stage diagnosis and limited access to treatment [1,3]. Regardless of regional survival outcomes, these treatments are frequently accompanied by a wide range of persistent physiological and psychological complications. Notably, the growing population of breast cancer survivors has shifted clinical priorities beyond survival toward long-term quality of life, highlighting the need to address treatment-related sequelae that extend well into survivorship [5].

These treatment-related sequelae span both physiological and psychological domains. Physiologically, survivors commonly experience lymphedema, chemotherapy-induced peripheral neuropathy, cardiotoxicity, accelerated bone mineral density loss, and reduced cardiorespiratory fitness [4,5]. Among psychological complications, anxiety represents one of the most prevalent and clinically significant symptoms, with levels commonly defined as Hospital Anxiety and Depression Scale (HADS) scores ≥8 (indicating possible clinical anxiety) or ≥11 (indicating probable clinical anxiety) [6], affecting approximately 40–54% of breast cancer patients and often persisting throughout the survivorship period [7]. Moreover, elevated anxiety levels are closely associated with increased fear of cancer recurrence (FCR) and have been linked to reduced adherence to adjuvant therapies (particularly endocrine therapy) as well as lower engagement with recommended follow-up care, factors that may indirectly influence long-term clinical outcomes [8].

Given these multidimensional consequences, the management of anxiety has become a central component of comprehensive cancer care. In this context, international clinical guidelines, including those from the American Society of Clinical Oncology (ASCO) and the National Comprehensive Cancer Network (NCCN), increasingly emphasize non-pharmacological interventions, particularly structured exercise, as a key strategy for improving psychological well-being [9,10]. Conventional physical exercise (PE), typically encompassing aerobic and resistance training, has consistently demonstrated beneficial effects on fatigue, anxiety, and depressive symptoms across cancer populations [11,12]. In parallel, mind–body exercise (MBE) modalities—such as yoga, Tai Chi, Qigong, and related practices—have gained growing attention for their capacity to regulate autonomic function and promote emotional well-being through the integration of movement, breath control, and mindfulness [13,14]. For the purposes of this review, PE refers to interventions focused on physiological conditioning—encompassing aerobic, resistance, and multicomponent training programs. The MBE category includes interventions in which movement is integrated with breath regulation, meditative attention, or mindfulness as a core component, including yoga, Tai Chi, Qigong, Baduanjin, and dance/movement therapy, together with a relaxation program incorporating progressive muscle relaxation, breathing and guided imagery. Although practices such as yoga and Tai Chi involve physical movement and can confer cardiorespiratory benefits, they were classified as MBE in this review because their structured integration of breathing technique and meditative focus represents a defining component, consistent with prior classification schemes in the oncology exercise literature [15,16].

Recent systematic reviews and meta-analyses (2023–2025) have consistently demonstrated that exercise interventions significantly reduce anxiety among breast cancer patients. Network meta-analyses indicate moderate overall effects (SMD ≈ −0.50), while both conventional PE and MBE interventions independently show comparable anxiolytic benefits [15,16,17]. In addition to anxiety reduction, these interventions have been associated with improvements in depression, fatigue, FCR, and overall quality of life. However, despite this growing body of evidence, important limitations remain. For example, Li et al. (2025) [16] focused exclusively on MBE interventions in breast cancer survivors, whereas other reviews evaluated PE modalities in isolation or pooled heterogeneous exercise interventions into broad categories without direct comparison [15,17]. Consequently, the relative effectiveness of PE and MBE has not been comprehensively evaluated within a single analytical framework, leaving uncertainty regarding potential differences between exercise modalities.

As a result, the relative effectiveness of different exercise modalities within a unified analytical framework remains unclear. Furthermore, findings from individual randomized controlled trials (RCTs) remain inconsistent, and key intervention parameters—including frequency, intensity, supervision, and timing—are still not well established. 

To address these gaps, the present study aimed to systematically review and meta-analyze controlled trials investigating the effects of both PE and MBE on anxiety in breast cancer patients across the cancer care continuum, encompassing individuals undergoing active oncological treatment (e.g., chemotherapy, radiotherapy, or surgery) as well as post-treatment survivors. The scope included conventional exercise, movement-based mind–body practices and a relaxation program.

This meta-analysis integrates multiple intervention modalities within a single analytical framework and compares their study-level effects. In addition to estimating the overall impact of interventions, subgroup analyses were conducted to explore potential differences according to intervention modality and clinical context. By doing so, this study seeks to provide clinically relevant evidence to inform individualized interventions in breast cancer care.

## 2. Methods

### 2.1. Reporting Framework and Study Records

**Figure 1:**
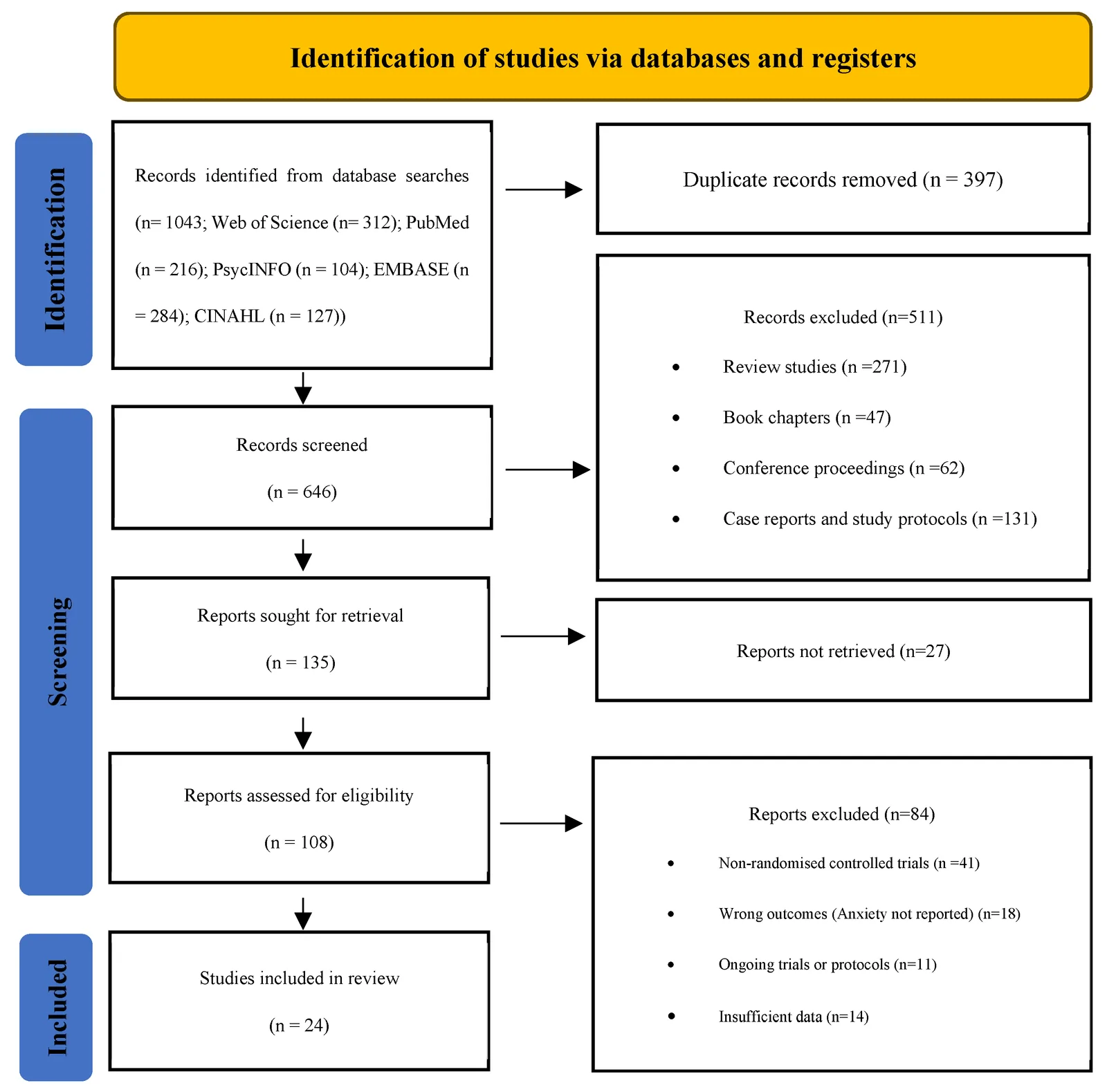
PRISMA 2020 flow diagram of the study selection process. The 24 included reports represent 25 independent trials; 22 trial estimates from 21 reports entered the quantitative synthesis.

This systematic review and meta-analysis was reported using the Preferred Reporting Items for Systematic Reviews and Meta-Analyses (PRISMA) 2020 framework [18]. A PRISMA flow diagram illustrating the study selection process is presented in Figure 1. To enhance methodological transparency and minimize the risk of selective reporting bias, Source reports are available in the public Open Science Framework (OSF) project at (https://osf.io/ax9be) (accessed on 10 July 2026). 

### 2.2. Eligibility Criteria

Study selection was conducted according to inclusion and exclusion criteria based on the PICOS (Population, Intervention, Comparison, Outcomes, Study Design) framework.

Population: Eligible studies included adult women (≥18 years) diagnosed with breast cancer (stage 0–IV). Both patients undergoing active oncological treatment (e.g., chemotherapy, radiotherapy, or surgery) and post-treatment survivors were considered. Studies including mixed or unspecified stages were included if the majority of participants had breast cancer. We acknowledge that breast cancer stages 0 (DCIS) and IV (metastatic disease) differ substantially in prognosis, treatment intent (curative vs. palliative), and psychological burden; this heterogeneity was explored through subgroup analyses by diagnostic stage and is further discussed in the Section 7. One report enrolled stages I–IV; none exclusively represented metastatic disease.

Intervention: Eligible interventions consisted of structured programs, categorized as either conventional PE (e.g., aerobic, resistance, or multicomponent training) or MBE (e.g., yoga, Tai Chi, Qigong, Baduanjin, or dance/movement therapy). Multimodal interventions incorporating movement-based or relaxation components were included if they contained a clearly defined PE or MBE component. The relaxation program of Jose and Cacodcar [19], comprising progressive muscle relaxation, breathing and guided imagery, was included in the MBE category. The multimodal exercise program of Zhang et al. [20] was categorized as PE.

Comparison: Control conditions included usual care, wait-list control, or attention/education-based interventions. Studies comparing two active exercise interventions without a non-exercise control group were excluded. 

Outcomes: Studies were required to report anxiety outcomes assessed using validated psychometric instruments, including but not limited to the Hospital Anxiety and Depression Scale—Anxiety subscale (HADS-A), State–Trait Anxiety Inventory (STAI), Beck Anxiety Inventory (BAI), Generalized Anxiety Disorder-7 (GAD-7), Self-Rating Anxiety Scale (SAS), and Profile of Mood States (POMS) tension/anxiety. Sufficient statistical data were required to calculate standardized effect sizes and their variances. One endpoint per independent trial was selected, ordinarily the first assessment at completion of the intervention. Reports without a defensible quantitative estimate were synthesized narratively.

Study Design: Controlled intervention studies were included. Observational studies, conference abstracts, reviews, case series, and case reports were excluded. Most reports described randomized controlled trials. Shehab et al. [21] described a quasi-experimental design while also stating that participants were randomly divided; the random sequence and allocation procedures were unclear. This report was retained as a controlled study with uncertain randomization. The evidence base also included one full-text preprint [19].

### 2.3. Search Strategy

A comprehensive and systematic literature search was conducted across five electronic databases: Web of Science, PubMed, EMBASE, PsycINFO, and CINAHL. The search covered records from database inception through April 2026.

Search terms were combined using Boolean operators (AND, OR). The search strategy incorporated three key domains: (1) breast cancer, (2) exercise interventions, and (3) anxiety outcomes. A representative search string was used as follows: (“breast cancer” OR “breast neoplasm*”) AND (“exercise” OR “physical activity” OR “aerobic training” OR “resistance training” OR “mind–body” OR “yoga” OR “tai chi” OR “qigong”) AND (“anxiety” OR “psychological distress”). The search was limited to articles published in English. Conference abstracts, dissertations, and theses were excluded. Full-text preprints were eligible when they provided sufficient methodological detail to assess study quality and contained extractable outcome data. The database search yielded a total of 1043 records, including Web of Science (n = 312), PubMed (n = 216), EMBASE (n = 284), PsycINFO (n = 104), and CINAHL (n = 127).

### 2.4. Statistical Analysis

All statistical analyses were performed using R software (version 4.5.1; R Foundation for Statistical Computing, Vienna, Austria) with the *metafor* package [22]. A random-effects meta-analysis using restricted maximum likelihood (REML) estimation was conducted. Anxiety outcomes were assessed using validated psychometric instruments reported by the included studies, including HADS-A, STAI, BAI, GAD-7, SAS, and the anxiety-related subscale of POMS. Because these instruments use different scoring systems and ranges, outcomes were pooled using standardized mean differences (Hedges’ g), which standardize treatment effects by the pooled standard deviation and allow comparison across different anxiety measures [23,24]. For raw endpoint data, Hedges’ g was calculated as the intervention-minus-control mean divided by the pooled within-group standard deviation (SD), with the exact small-sample correction. Negative values favor the intervention. Means and SDs were paired with their corresponding analysis-group sample sizes. Imputed intention-to-treat summaries were used when explicitly reported. HADS-A was selected when available; state rather than trait anxiety was selected for STAI reports. Independent endpoint contrasts were distinguished from the source studies’ adjusted or longitudinal tests. Although standardization facilitates quantitative synthesis, differences in the specific anxiety constructs assessed (e.g., state versus trait anxiety) may contribute to between-study heterogeneity.

Cadmus et al. [25] contributed two independent cohorts, IMPACT and YES. The two eligible exercise arms in Lavín-Pérez et al. [26] were combined using their sample sizes, means and within- and between-arm sums of squares, with the control counted once. The adjusted mean differences and confidence intervals reported by Travier et al. [27] and Rogers et al. [28] were standardized by pooled baseline SDs and entered using generic inverse variance. Their standard errors were approximated from the reported confidence intervals, with an additional variance term for uncertainty in the standardizer. The Ying et al. [29] estimate was derived from the reported independent-samples t statistic and group counts, assuming a pooled-variance *t* test. 

Heterogeneity was evaluated using Cochran’s Q test and the I^2^ statistic, with τ^2^ describing between-trial variance [30]. A random-effects model using REML estimation was applied. Confidence intervals and tests used modified Hartung–Knapp inference with the residual scale bounded below by one and k−1 degrees of freedom. The 95% prediction interval used a t distribution with k−2 degrees of freedom. Clinical diversity and the dispersion of study effects informed interpretation of heterogeneity and the assessment of inconsistency within the Grading of Recommendations Assessment, Development, and Evaluation (GRADE) framework.

Seven separate exploratory univariable meta-regressions evaluated baseline anxiety, mean age, program duration, exercise frequency, total sample size, session duration and cumulative prescribed dose. Each model used REML and modified Hartung–Knapp inference, with the variance multiplier bounded below at one and *t* tests with k − 2 degrees of freedom. Models used complete cases for each moderator, without imputation. Baseline anxiety was the intervention-arm mean HADS-A score immediately before intervention; raw baseline scores from different instruments were not combined. Mean age was the reported study mean or a sample-size-weighted mean across the represented arms; medians and age categories were not converted. Total sample size represented the reported analysis population, counting each shared control once. The combined exercise arms in the three-arm trial were represented by their weighted mean.

Program duration was expressed in weeks, with six months treated as 26 weeks. Frequency and session duration described the prescribed target program, including specified home practice; changing schedules and different session types were weighted by their duration or frequency, respectively. Unspecified component times and session-length ranges were left missing. Cumulative dose was the nominal target weekly minutes multiplied by program duration, using stated minimum targets where applicable. This measure does not quantify achieved exercise, progression or adherence. R^2^ was the proportional reduction in REML τ^2^ relative to an intercept-only model fitted to the same complete-case subset, bounded below at zero. *p* values were reported without adjustment and interpreted as exploratory; the Bonferroni threshold for seven tests was 0.05/7 = 0.0071.

Subgroup analyses were performed to explore potential differences according to intervention modality, treatment phase and diagnostic stage. Subgroup summaries used separate within-group REML estimates; formal comparisons used categorical meta-regression with a common residual τ^2^ and modified Hartung–Knapp F tests. No modality-by-phase interaction was fitted. Influence diagnostics, including a Baujat plot, were applied to identify studies contributing disproportionately to heterogeneity [31]. The plot describes contributions to Q and standardized influence relative to the common-effect reference used in this diagnostic. Leave-one-out analysis omitted each independent trial estimate in turn, re-estimating REML heterogeneity and applying modified Hartung–Knapp inference (21 estimates; 20 degrees of freedom). Funnel plots and Egger’s regression test [32] were used to assess small-study effects; their interpretation considered substantial heterogeneity and the mathematical relationship between standardized effects and their standard errors. Rank-correlation testing [33] and fail-safe N approaches [34,35] were not applied. Trim-and-fill [36] used the L0 estimator and REML during trimming and final pooling, with modified Hartung–Knapp inference. The REML slope on standard error selected the imputation side. The procedure assumes one-sided missingness and cannot establish or exclude publication bias. Methodological quality was assessed using the Physiotherapy Evidence Database (PEDro) scale [37,38]. 

Finally, the overall certainty of evidence for the anxiety outcome was evaluated using the GRADE approach [39]. Certainty ratings were determined at the outcome level (anxiety) and considered the five standard GRADE domains: risk of bias, inconsistency, indirectness, imprecision, and publication bias.

## 3. Results

The database search identified a total of 1043 records. After removing 397 duplicates, 646 records remained for title and abstract screening, of which 511 were excluded. A total of 135 reports were sought for retrieval; 27 were not retrieved. A total of 108 articles were assessed for full-text eligibility, and 84 were excluded due to non-randomized designs (n = 41), absence of anxiety-related outcomes (n = 18), ongoing trials or protocols (n = 11), and insufficient data for effect size calculation (n = 14). Ultimately, 24 reports met the inclusion criteria. As previously mentioned, Cadmus et al. [25] reported two independent trials, yielding 25 trials across the 24 reports. Twenty-two estimates from 21 reports were included in the meta-analysis.

Three reports were synthesized narratively. Barcelos de Souza et al. [40] evaluated sequential dance programs in the same intervention cohort; the free-dance and control assessments were not contemporaneous, and reported dispersions were standard errors. Larkey et al. [41] reported adjusted marginal means and confidence intervals without sufficient information to construct a compatible between-group standardized contrast with its variance. Drozd et al. [42] reported endpoint HADS-A means and SDs but did not establish the corresponding respondent counts. These reports were therefore excluded from quantitative pooling.

The included studies covered the breast cancer care continuum, including both post-treatment survivors and patients undergoing active treatment such as chemotherapy and radiotherapy. Most reports concerned early/nonmetastatic disease. Only one study (Zhang et al., 2023) [20] included a mixed sample spanning stages I–IV, and no study exclusively enrolled patients with metastatic (stage IV) disease. The quantitative synthesis included 14 PE estimates and eight MBE estimates; 12 estimates concerned active treatment and ten concerned survivorship. PE protocols primarily included aerobic, resistance, or multicomponent training, while MBE interventions involved practices integrating movement, breath control, mindfulness or relaxation. Endpoints ranged from two weeks to approximately six months. Anxiety outcomes were assessed using validated instruments, most commonly HADS-A, alongside STAI, BAI, GAD-7, SAS, and POMS. As these instruments capture related but distinct constructs, pooling results across different scales may introduce measurement heterogeneity and affect the comparability of effect sizes. To address this, standardized effect sizes were used; however, some scale-specific nuances may not be fully reflected. Methodological quality assessed using the PEDro scale is presented in Table 1, and certainty of evidence assessed using GRADE is presented in Table 2. The characteristics of included studies and their analysis populations are presented in Table 3.

The mean PEDro score of the included reports was 5.92/10. Uncertainty in allocation methods, unblinded self-reported outcomes, missing data and differing analysis populations limit confidence in the estimates.

According to Table 2, the overall certainty of evidence for anxiety outcomes was rated as very low. This grading reflects concerns related to risk of bias, substantial heterogeneity and imprecision across the included studies. Funnel asymmetry also raises concern about small-study effects without establishing publication bias. Although the pooled effect indicated a statistically significant reduction in anxiety, the variability in study design and intervention characteristics limits the strength of the conclusions. The characteristics of included studies are presented in Table 3.

### 3.1. Pooled Effect and Heterogeneity

**Figure 2:**
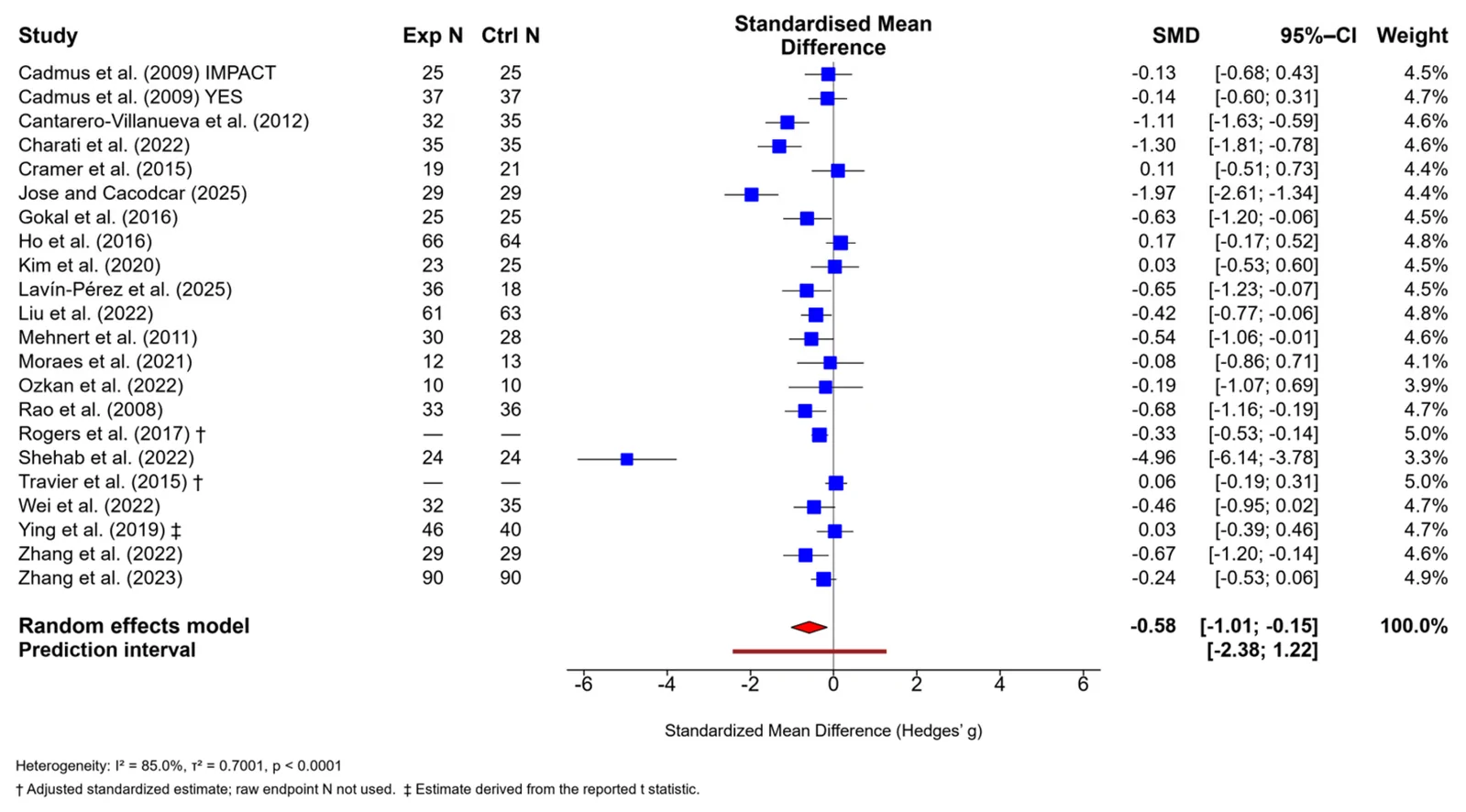
Forest plot of intervention effects on anxiety. Blue squares represent trial estimates, black lines their 95% confidence intervals, the red diamond the random-effects summary, and the red bar the prediction interval. Weights refer to the REML model. Individual intervals use normal approximation; pooled intervals use modified Hartung–Knapp inference. † Adjusted standardized contrast; ‡ t-derived estimate. No raw endpoint count is assigned to an adjusted estimate.

The forest plot in Figure 2, encompassing 22 independent trial estimates, demonstrates a statistically significant overall effect of interventions on reducing anxiety symptoms among breast cancer patients (SMD = −0.58, 95% CI [−1.01, −0.15], *p* = 0.011). The magnitude of the standardized effect can be interpreted as moderate, although it does not establish a clinically important change on an individual anxiety scale. The estimated heterogeneity was substantial (τ^2^ = 0.7001, I^2^ = 85.0%). Cochran’s Q test was statistically significant (Q(21) = 139.90, *p* < 0.0001), indicating considerable variability in effect sizes across the included trials (Table 4). The 95% prediction interval was −2.38 to 1.22, indicating uncertainty about the direction and magnitude of effects across comparable settings.

### 3.2. Small-Study Effects and Trim-and-Fill

**Figure 3:**
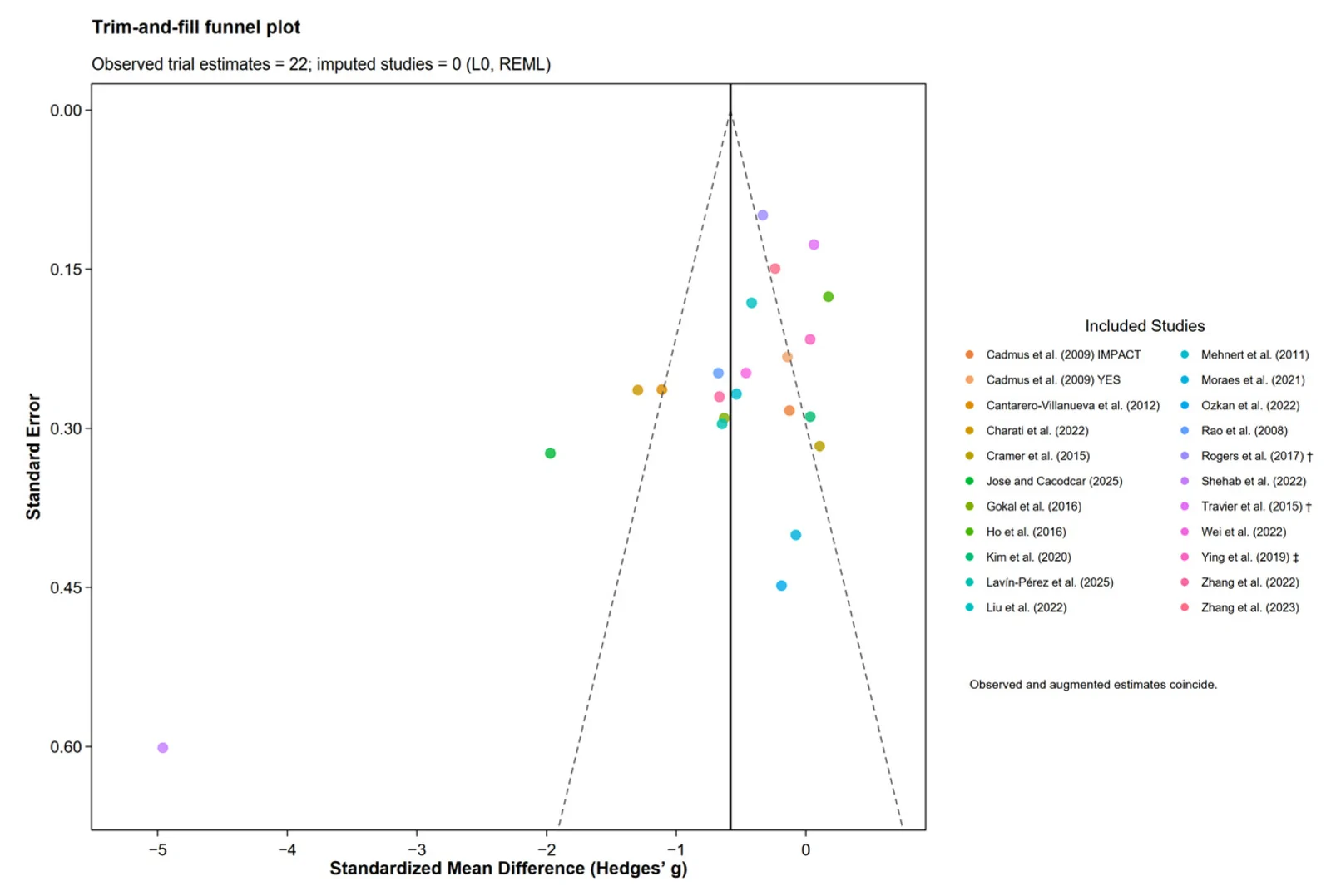
Trim-and-fill funnel plot of the trial estimates. The solid line marks the pooled effect; dashed lines mark approximate 95% limits around it. Colors identify the trial estimates. Trim-and-fill (L0, REML) retained 22 observed trial estimates and imputed 0 studies; the observed and augmented plots coincide. † Adjusted standardized contrast; ‡ t-derived estimate.

Visual inspection of the funnel plot (Figure 3) suggested asymmetry, which was statistically significant according to Egger’s regression test (t(20) = −2.48, *p* = 0.022), indicating potential small-study effects. The regression intercept was −3.052 (SE 1.233; Table 5). These findings suggest that potential publication bias cannot be ruled out. Trim-and-fill estimated no missing studies on the right side of the funnel (L0 = 0). The pooled estimate therefore remained −0.58 (95% CI [−1.01, −0.15], *p* = 0.011), and the augmented funnel plot coincided with the observed plot. 

### 3.3. Contributions to Heterogeneity and Influence

**Figure 4:**
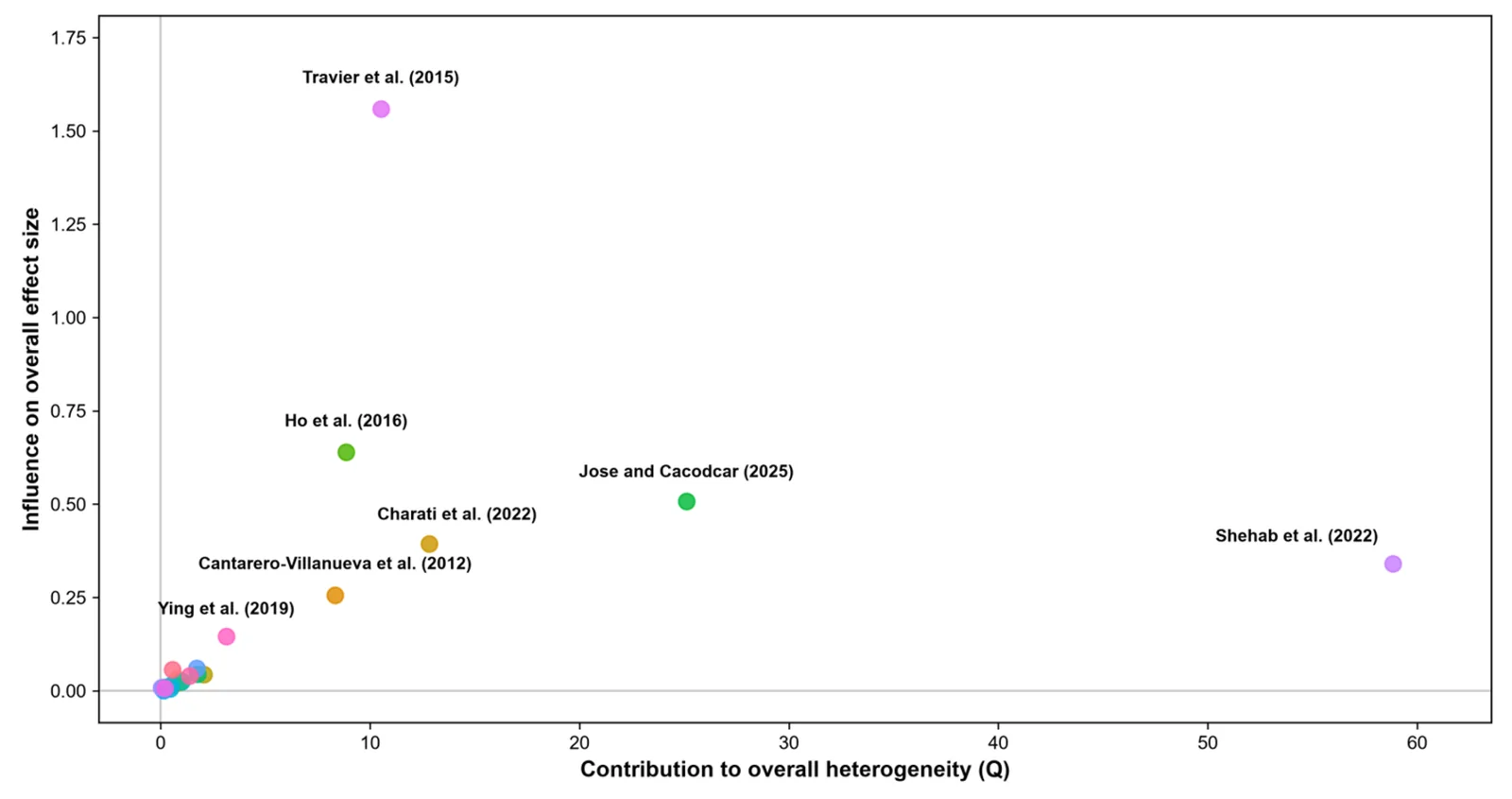
Baujat plot. The horizontal axis shows each trial’s contribution to Q. The vertical axis shows standardized squared influence relative to the common-effect reference. Selected influential trials are labelled.

The Baujat plot (Figure 4) was employed to identify studies disproportionately contributing to overall heterogeneity and influencing the common-effect reference used in this diagnostic. The visualization identified Shehab et al. (2022) [21] as the primary contributor to overall heterogeneity (highest Q-contribution), whereas Travier et al. (2015) [27] exhibited the most substantial standardized influence on the common-effect reference. Jose and Cacodcar (2025) [19] and Charati et al. (2022) [44] were the next-largest contributors to Q, while Ho et al. (2016) [47] also showed a combined impact on heterogeneity and influence. 

### 3.4. Subgroup Analyses

**Figure 5:**
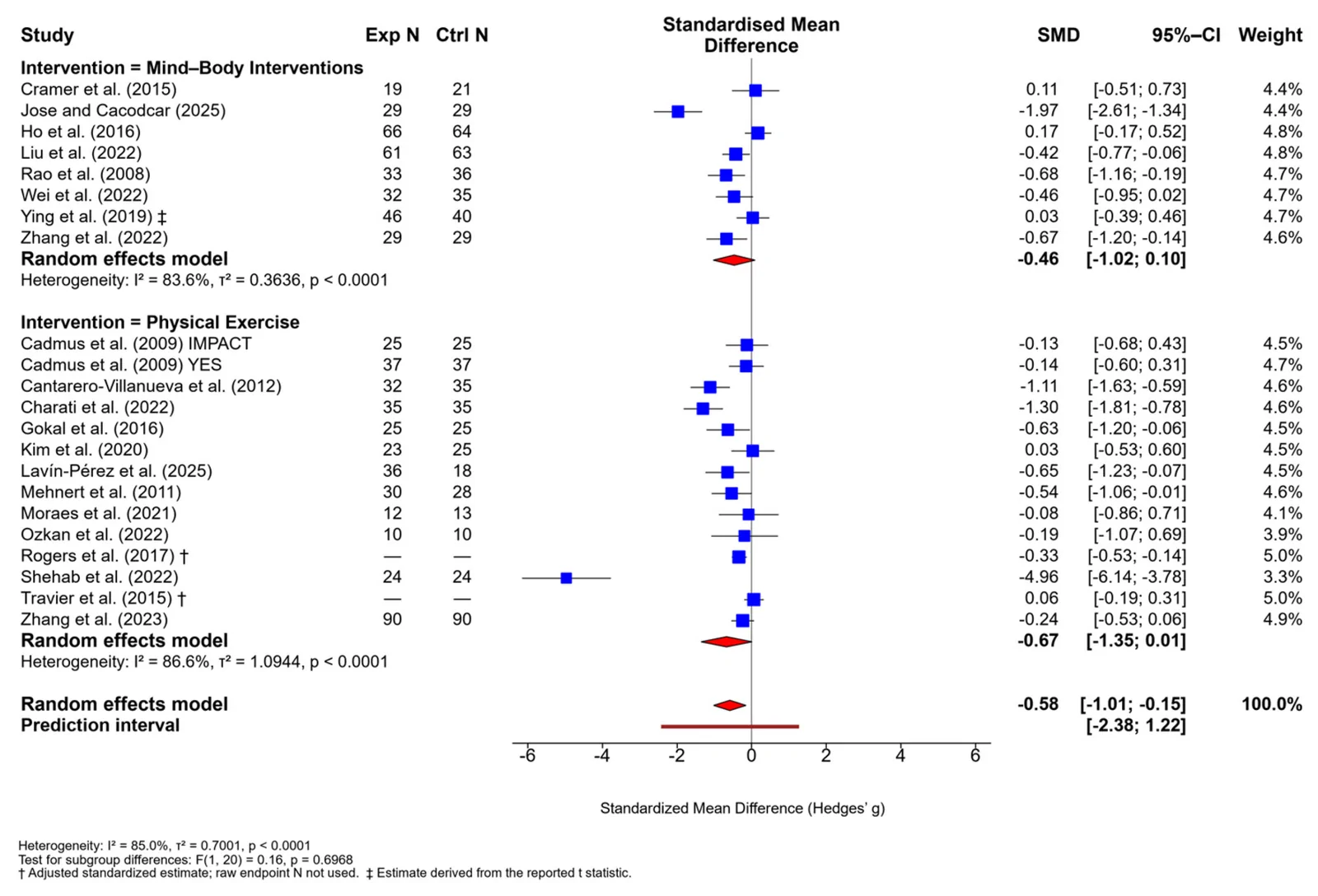
Forest plot by intervention modality. PE, physical exercise; MBE, mind–body intervention, including the relaxation program. Subgroup summaries use separate REML estimates; the formal subgroup test uses a common residual variance. Weights are percentages of the overall random-effects model. Symbols and derived estimates are defined in Figure 2.

A subgroup meta-analysis was performed (Figure 5) to compare the effects of mind–body interventions (k = 8) and physical exercise (k = 14) on anxiety symptoms among breast cancer patients. While both mind–body interventions (SMD = −0.46; 95% CI [−1.02, 0.10]) and physical exercise (SMD = −0.67; 95% CI [−1.35, 0.01]) favoured intervention numerically, both confidence intervals included no effect. The test for subgroup differences revealed no statistically significant difference between the two modalities (F(1,20) = 0.16, *p* = 0.697). Substantial heterogeneity was observed within both subgroups.

**Figure 6:**
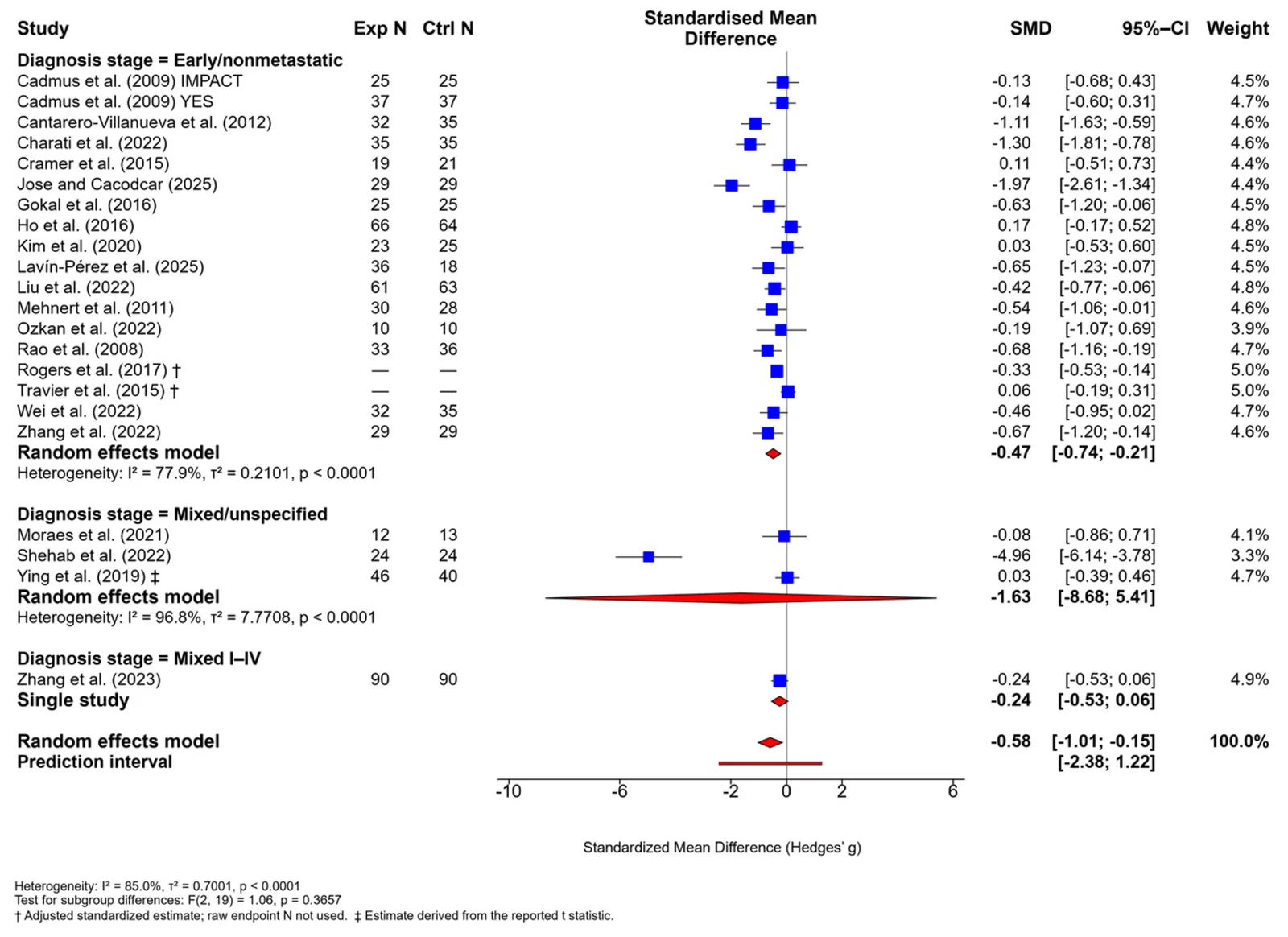
Forest plot by diagnostic stage. The mixed stages I–IV category contains one trial; its interval is the individual normal-approximation interval. Other subgroup summaries use REML and modified Hartung–Knapp inference. Symbols, derived estimates and weights are defined in Figure 5 and Figure 6.

Subgroup analysis by diagnostic stage (Figure 6) shows that interventions yielded a significant reduction in anxiety for the early/nonmetastatic group (k = 18; SMD = −0.47; 95% CI [−0.74, −0.21]), while the mixed/unspecified group showed a larger but non-significant point estimate (k = 3; SMD = −1.63; 95% CI [−8.68, 5.41]). One trial enrolled stages I–IV and gave an SMD of −0.24 (95% CI [−0.53, 0.06]). The test for subgroup differences was not statistically significant (F(2,19) = 1.06, *p* = 0.366). 

**Figure 7:**
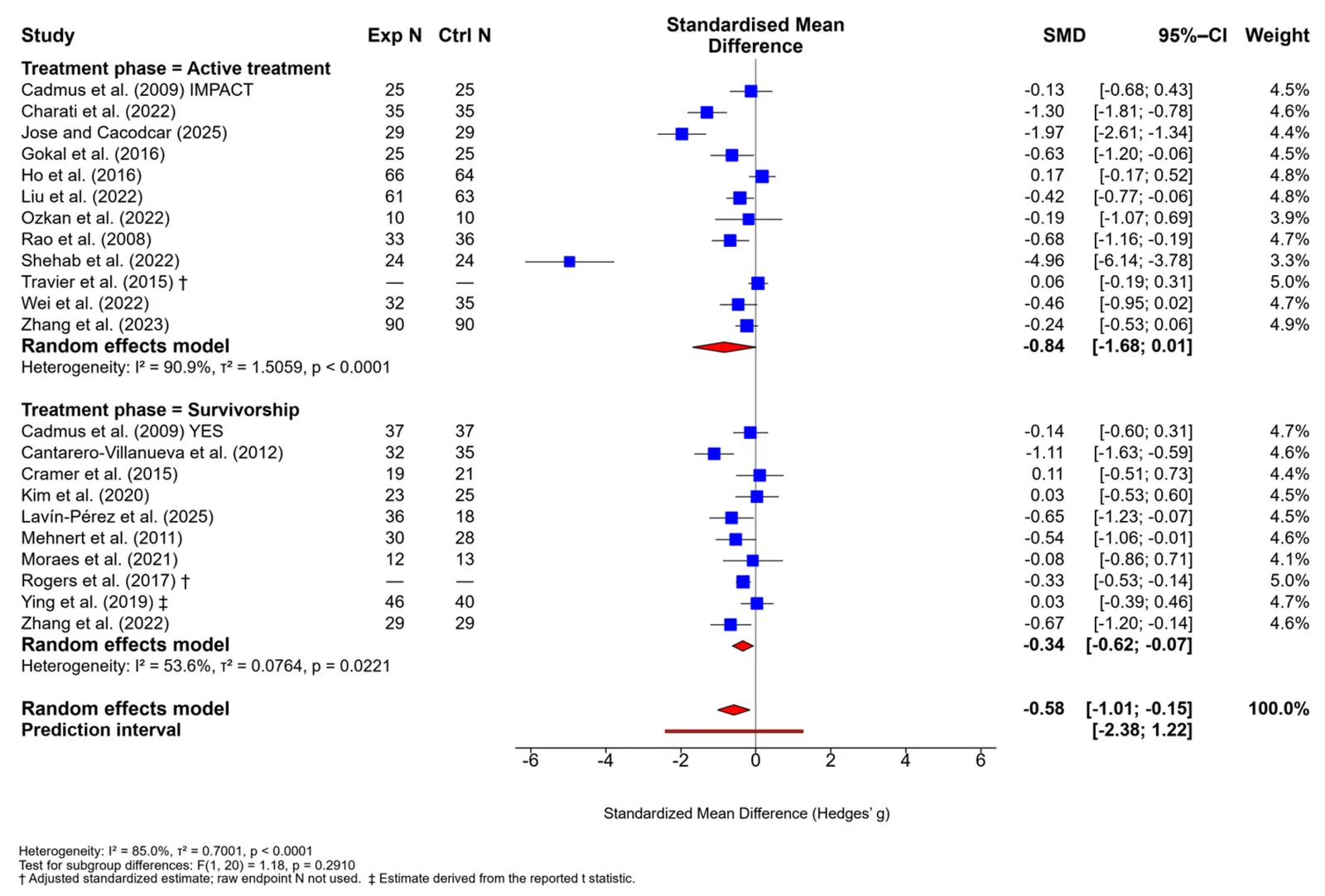
Forest plot by treatment phase. IMPACT and YES are independent trials reported by Cadmus et al. [25]. Subgroup summaries use separate REML estimates; the formal comparison uses a common residual variance. Symbols, derived estimates and weights are defined in Figure 5 and Figure 6.

Subgroup analysis by treatment phase (Figure 7) indicates that the survivorship group demonstrated a significant reduction in anxiety (k = 10; SMD = −0.34; 95% CI [−0.62, −0.07]), whereas the active-treatment group showed a larger but non-significant effect (k = 12; SMD = −0.84; 95% CI [−1.68, 0.01]). Although the effect size appeared larger during active treatment, the difference between these phases did not reach statistical significance (F(1,20) = 1.18, *p* = 0.291). 

### 3.5. Meta-Regression Analyses

Meta-regression analyses for continuous moderators are presented in Table 6. Higher intervention-group baseline HADS-A scores were associated with more negative effect estimates (k = 13; β = −0.2551 per point, 95% CI [−0.4535, −0.0566], *p* = 0.0164), with an estimated R^2^ of 46.45%. This association was nominally significant but did not meet the threshold accounting for seven tests (*p* < 0.0071). Program duration was not statistically significant (β = 0.0504 per week, 95% CI [−0.0056, 0.1065], *p* = 0.0753; R^2^ = 17.18%). Mean age, frequency, total sample size, session duration and cumulative prescribed dose were also not statistically significant (all *p* ≥ 0.1606). 

### 3.6. Leave-One-Out Analysis

**Figure 8:**
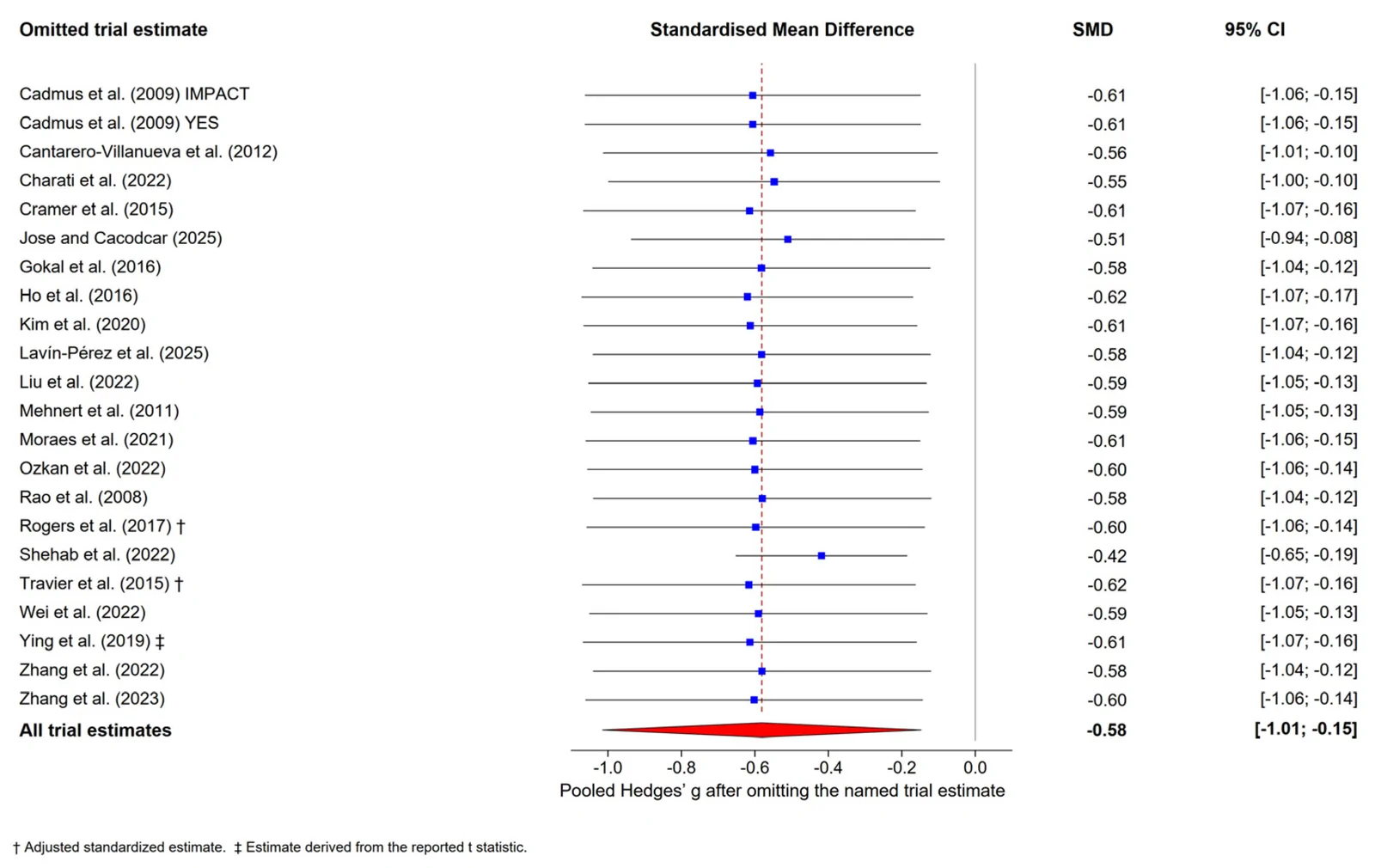
Leave-one-out analysis. Each blue square shows the pooled effect after omitting the named independent trial estimate; black lines show modified Hartung–Knapp 95% confidence intervals. REML heterogeneity was re-estimated at each omission. The red diamond and dashed red line mark the full-model pooled effect; the grey line marks no effect. † Adjusted standardized contrast; ‡ t-derived estimate.

Sequential omission of each of the 22 independent trial estimates yielded pooled effects ranging from −0.62 to −0.42; all 95% confidence intervals excluded zero (*p* range 0.0013–0.0213; Figure 8). Omission of Shehab et al. [21] produced the largest attenuation, giving an SMD of −0.42 (95% CI [−0.65, −0.19], *p* = 0.0013), with I^2^ = 75.2% and τ^2^ = 0.1850. Thus, the direction and statistical significance of the pooled result did not depend on any single trial estimate, although its magnitude and heterogeneity were influenced by individual trials.

## 4. Discussion

The findings suggest an average reduction in anxiety, with a moderate overall standardized effect size (SMD = −0.58, 95% CI [−1.01, −0.15]), supporting further evaluation of physical and mind–body interventions for psychological symptom management in breast cancer patients. However, the magnitude of a standardized effect does not establish a clinically meaningful change on a particular anxiety scale, and the 95% prediction interval (−2.38 to 1.22) indicates uncertainty about the direction and magnitude of effects across comparable settings. While the present meta-analysis focused exclusively on anxiety outcomes, prior systematic reviews and meta-analyses have similarly reported that PE and MBE interventions are associated with broader improvements in health-related quality of life (HRQoL), including reductions in depressive symptoms and cancer-related fatigue [15,17], suggesting that the anxiolytic benefits observed here may occur alongside other psychological and physical benefits not directly assessed in the present analysis. The mean PEDro score (5.92/10) and the “very low” GRADE certainty rating reflect different dimensions of evidence appraisal. PEDro evaluates methodological features of individual trials, focusing on criteria such as randomization, allocation procedures, baseline comparability, and completeness of outcome reporting. In contrast, GRADE assesses the certainty of the pooled effect estimate across the entire body of evidence and considers five domains: risk of bias, inconsistency, indirectness, imprecision, and publication bias.

In the present review, the certainty of evidence was downgraded because of concerns about risk of bias, inconsistency and imprecision. First, the difficulty of blinding participants and personnel in exercise-based interventions contributes to risk-of-bias assessments, alongside unclear allocation methods, missing outcome data and differing analysis populations. Second, substantial between-study heterogeneity (I^2^ = 85.0%) reduced confidence in the consistency of the observed effects. Third, the confidence interval spanned small to large, standardized effects. These considerations are not fully captured by PEDro scores, which are assigned at the individual report level. Therefore, the overall certainty of the pooled estimate remains very low, indicating that the true effect may differ meaningfully from the estimated effect size. We should therefore interpret the magnitude of the pooled effect with appropriate caution.

Subgroup analyses further explored intervention effects across different clinical contexts. A reduction in anxiety was observed during survivorship (SMD = −0.34; 95% CI [−0.62, −0.07]), whereas the larger estimate during active treatment did not reach statistical significance (SMD = −0.84; 95% CI [−1.68, 0.01]). Although the magnitude of the effect was numerically greater during the active treatment phase, the difference between phases was not statistically significant (F(1,20) = 1.18, *p* = 0.291). This does not establish consistent efficacy across the cancer continuum. Similarly, the comparison across diagnostic stages was inconclusive. While patients with early or nonmetastatic disease demonstrated a reduction in anxiety (SMD = −0.47; 95% CI [−0.74, −0.21]), the mixed or unspecified group showed a larger but non-significant effect (SMD = −1.63; 95% CI [−8.68, 5.41]). The between-category test was not statistically significant (F(2,19) = 1.06, *p* = 0.366). The wide confidence interval in the mixed or unspecified group reflects substantial uncertainty and does not establish a clinical difference between diagnostic categories.

PE, particularly aerobic and resistance training, may exert anxiolytic effects through multiple physiological and psychological pathways [9,15]. In addition, supervised exercise programs may support psychological well-being by improving physical fitness and enhancing self-efficacy [9,28]. However, during periods of intensive treatment such as chemotherapy, treatment-related fatigue and reduced functional capacity highlight the importance of individualized exercise prescription. The included studies did not consistently measure mediators, and the present meta-analysis did not test the mechanisms underlying the observed anxiety effects.

MBE, including yoga, Tai Chi, and Qigong, integrates controlled breathing, mindful movement, and meditative focus [13,14]. These practices may promote a relaxation response and support emotional well-being. Beyond physiological pathways, reducing social isolation and enhancing coping capacity are plausible psychosocial mechanisms. These explanations remain hypotheses within the present synthesis; biological plausibility does not establish comparative efficacy between intervention categories.

Overall, both PE and MBE favored intervention numerically, but their subgroup confidence intervals included no effect. The test for subgroup differences was inconclusive, and the absence of a statistically significant difference does not establish equivalence. The stage-related findings derive from independently conducted subgroup analyses rather than from a formal modality-by-stage interaction test and therefore do not constitute evidence of differential efficacy between PE and MBE across treatment phases. The low-impact and adaptable nature of some MBE programs may plausibly facilitate participation when fatigue and reduced functional capacity are present, while structured aerobic and resistance training may support physical reconditioning and psychological well-being [9,17,28]. These clinical considerations warrant confirmation through prospective head-to-head comparative trials before any inference regarding differential efficacy can be drawn.

The nominal association between higher baseline HADS-A scores and larger anxiety reductions suggests that initial symptom burden may warrant further investigation. However, it did not meet the threshold accounting for seven moderator tests, and the study-level comparison cannot separate symptom burden from differences in populations, interventions, baseline group imbalance or regression to the mean. The absence of statistically significant associations for the remaining continuous moderators does not demonstrate that these characteristics are unimportant; confidence intervals were wide, and some models included only 13 estimates.

Taken together, the findings indicate a possible beneficial role of physical and mind–body interventions in breast cancer care. However, substantial heterogeneity (I^2^ = 85.0%) limits the precision and interpretability of pooled estimates and reflects clinical and methodological diversity. Potential contributors include variation in intervention type (e.g., aerobic vs. resistance vs. yoga vs. Tai Chi), intensity and supervision format (supervised vs. home-based vs. mixed), intervention duration, treatment phase at the time of intervention, and the use of different anxiety measurement instruments (HADS-A, STAI, BAI, SAS, GAD-7, and POMS). Instrument-related measurement heterogeneity remains a plausible contributor, as different scales assess related but not identical dimensions of anxiety. The continuous meta-regressions did not establish program duration, frequency, session duration or prescribed dose as moderators. The Baujat plot identified studies contributing disproportionately to heterogeneity and to the common-effect reference used in the influence diagnostic. Leave-one-out analysis retained a negative pooled effect with confidence intervals excluding zero after every omission, although removing Shehab et al. [21] attenuated the effect and reduced heterogeneity. Furthermore, evidence of small-study effects (Egger’s test, t(20) = −2.48, *p* = 0.022) warrants cautious interpretation. Funnel asymmetry cannot distinguish publication bias from heterogeneity or the relationship between standardized effects and their estimated standard errors. Trim-and-fill imputed no studies, but this result does not demonstrate absence of publication bias, particularly given substantial heterogeneity. Earlier reviews have examined exercise during active treatment [56] and Tai Chi Chuan for quality of life and psychological well-being [57]. The broad scope, including a relaxation preprint and a study with uncertain randomization, further limits comparisons with reviews restricted to conventional exercise trials with established random allocation.

## 5. Practical Applications

Exercise should be prescribed as a structured, individualized, and clinically integrated intervention [9]. Patient tolerance, symptom burden, functional capacity, preferences and access to supervision should inform individualized decision-making. The present meta-analysis did not test an interaction between intervention modality (PE vs. MBE) and treatment phase (active treatment vs. survivorship), and the separate subgroup analyses provide no basis for preferring one category at a particular phase.

During intensive treatment phases (e.g., chemotherapy and radiotherapy), low-intensity MBE modalities such as Baduanjin may represent a reasonable option for patients with reduced tolerance to higher-intensity physical loads, where clinically appropriate [9,54]. This should be considered a plausible clinical option rather than an evidence-based superiority claim. Exercise interventions should complement appropriate assessment and management of anxiety; a favorable standardized mean difference does not measure remission or the proportion of patients achieving a clinically important improvement.

During the survivorship phase, structured and supervised multicomponent PE programs combining aerobic and resistance training may be valuable for restoring physical function and fitness, with potential downstream benefits for psychological well-being [9,28]. The present analysis does not establish that PE should be preferred over MBE during survivorship. Collectively, these considerations support a flexible, patient-centered approach to exercise selection within multidisciplinary cancer care, pending future trials designed to formally test modality-by-stage interactions.

## 6. Clinical Implications and Future Perspectives

Integrative oncology provides a framework in which exercise physiologists, oncologists, and psycho-oncology specialists can collaboratively design individualized, evidence-based care plans. From an implementation perspective, home-based programs such as Baduanjin delivered with video guidance, as employed in included trials [29,54], may offer an accessible option in resource-limited oncology settings. Supervised PE programs combining aerobic and resistance training may require access to trained exercise professionals and suitable equipment or facility space, depending on the program. These implementation considerations were not evaluated by the present meta-analysis. Future research should formally evaluate the comparative cost-effectiveness and implementation feasibility of these modalities, including patient-level costs, healthcare system resource utilization, and scalability across diverse clinical settings, to inform evidence-based resource allocation in integrative oncology programs.

Within this framework, assessment of anxiety using validated instruments (e.g., HADS, STAI) can inform referral for appropriate psychological and supportive care. Future research should prioritize multi-arm, head-to-head RCTs directly comparing different intervention modalities across clearly defined clinical stages. Such designs are essential to establish optimal intervention strategies and refine stage-specific exercise prescriptions. The integration of objective biomarkers, such as heart rate variability and inflammatory markers, may help elucidate mechanisms underlying PE and MBE interventions. Furthermore, future studies should incorporate standardized reporting of intervention characteristics and adherence outcomes to improve reproducibility and facilitate more precise dose–response analyses. Trials should clearly report allocation methods, prespecified anxiety outcomes, endpoint-specific group counts, raw means and SDs, adjusted contrasts with confidence intervals, and missing-data procedures. Multi-arm studies should provide sufficient information to combine eligible groups without counting a shared control twice.

## 7. Limitations

Several limitations should be acknowledged when interpreting these findings. First, the inherent difficulty of blinding participants in exercise-based interventions introduces a potential risk of performance bias [58], while unblinded self-reported outcomes, unclear allocation procedures and missing data further limit confidence. Second, substantial heterogeneity was observed across studies, likely reflecting variability in intervention characteristics (e.g., frequency, intensity, and type), treatment stages, and delivery formats. This heterogeneity limits precise interpretation of the pooled effect and does not establish a dose–response relationship. Third, the use of diverse self-reported anxiety assessment tools (e.g., HADS-A, BAI, POMS, SAS) necessitated standardized effect sizes, which may obscure subtle scale-specific differences. Fourth, adherence and compliance were inconsistently reported across studies, potentially influencing the magnitude of the observed effects. Fifth, the exclusion of some grey literature (e.g., dissertations and conference proceedings) and the restriction to English-language reports may have increased the risk of reporting bias; one full-text preprint was included. Evidence of small-study effects also reduces confidence, although funnel asymmetry is not diagnostic of publication bias. Furthermore, the inclusion of different breast cancer stages introduces clinical heterogeneity, as these stages differ substantially in prognosis, treatment intent, and psychological burden. Subgroup findings should be interpreted with caution given the limited number of studies in some categories, and no study exclusively enrolled patients with metastatic disease. The indirect comparisons do not establish individual-level effect modification, and no modality-by-phase interaction was tested. Continuous meta-regressions were exploratory, used different complete-case subsets and could not adjust simultaneously for confounding. Missing or ambiguous prescription details reduced the evidence available for session duration and dose; nominal target minutes do not measure completed exercise or its intensity. Multiple testing further limits interpretation of the baseline-anxiety association. Three reports could not contribute a comparable quantitative estimate. The synthesis combines unadjusted endpoint contrasts, explicitly reported imputed summaries, two adjusted effects standardized by baseline SDs, and one estimate derived from a t statistic. These represent different analysis populations and are not identical estimands. Approximation from rounded confidence intervals and the pooled-variance assumption for the t-derived estimate add uncertainty. The inclusion of a relaxation preprint and a controlled study with uncertain randomization extends the scope beyond conventional exercise RCTs. Finally, the analysis does not establish clinical importance on an individual anxiety scale.

## 8. Conclusions

This meta-analysis suggests that physical and mind–body interventions are associated with reduced anxiety on average in breast cancer patients. However, substantial heterogeneity and very low certainty limit confidence in the size and consistency of benefit. No statistically significant differences were observed between intervention modalities or treatment phases, and these findings do not establish superiority or equivalence. This study did not directly test differences in efficacy between PE and MBE across treatment phases; therefore, no formal inference regarding stage-specific superiority can be made based on the present analyses. The findings support further evaluation of individualized interventions within multidisciplinary oncology care. Prospective head-to-head randomized trials with complete outcome data are needed to establish comparative efficacy and inform intervention selection for specific patient groups.

## Figures and Tables

**Table 1:** PEDro assessments of the included reports.

Study	EC	I	II	III	IV	V	VI	VII	VIII	IX	X	Total
Barcelos de Souza et al. (2025) [40]	√	√	√	√	×	×	×	√	×	√	√	6
Cadmus et al. (2009) [25]	√	√	√	√	×	×	×	√	√	√	√	7
Cantarero-Villanueva et al. (2012) [43]	√	√	√	√	×	×	×	√	√	√	√	7
Charati et al. (2022) [44]	√	√	×	√	×	×	√	√	×	√	√	6
Cramer et al. (2015) [45]	√	√	√	√	×	×	×	√	√	√	√	7
Jose and Cacodcar (2025) [19]	√	√	√	√	×	×	×	√	×	√	√	6
Drozd et al. (2026) [42]	√	√	×	×	×	×	×	×	×	√	√	3
Gokal et al. (2016) [46]	√	√	×	√	×	×	×	√	√	√	√	6
Ho et al. (2016) [47]	√	√	√	√	×	×	×	√	√	√	√	7
Kim et al. (2020) [48]	√	√	×	√	×	×	×	√	×	√	√	5
Larkey et al. (2025) [41]	√	√	√	×	×	×	×	×	√	√	√	5
Lavín-Pérez et al. (2025) [26]	√	√	×	√	×	×	×	√	×	√	√	5
Liu et al. (2022) [49]	√	√	√	√	×	×	×	√	√	√	√	7
Mehnert et al. (2011) [50]	√	√	√	√	×	×	×	√	×	√	√	6
Moraes et al. (2021) [51]	√	√	×	×	×	×	×	√	×	√	√	4
Ozkan et al. (2022) [52]	√	√	√	√	×	×	√	√	×	√	√	7
Rao et al. (2008) [53]	√	√	√	√	×	×	×	×	×	√	√	5
Rogers et al. (2017) [28]	√	√	√	×	×	×	√	√	√	√	√	7
Shehab et al. (2022) [21]	√	√	×	√	×	×	×	×	×	√	√	4
Travier et al. (2015) [27]	√	√	√	√	×	×	√	√	√	√	√	8
Wei et al. (2022) [54]	√	√	×	√	×	×	×	√	√	√	√	6
Ying et al. (2019) [29]	√	√	√	√	×	×	√	√	×	√	√	7
Zhang et al. (2022) [55]	√	√	√	√	×	×	×	√	×	√	√	6
Zhang et al. (2023) [20]	√	√	×	√	×	×	×	√	×	√	√	5
Mean Score												5.92

Abbreviations: EC, eligibility criteria; I, random allocation; II, concealed allocation; III, baseline comparability; IV, blinding of subjects; V, blinding of therapists; VI, blinding of assessors; VII, outcome measures obtained from more than 85% of participants initially allocated to groups; VIII, intention-to-treat analysis; IX, between-group comparisons; X, reporting of point estimates and variability.√ indicates criterion fulfilled; × indicates criterion not fulfilled.

**Table 2:** Quality of Evidence (GRADE) assessment.

Certainty Assessment							No. of Patients		Effect	Certainty	Importance
No. of Studies	Study Design	Risk of Bias	Inconsistency	Indirectness	Imprecision	Other Considerations	Exercise	Control	Standardized (95% CI)		
22 estimates; 21 reports	Controlled trials ^a^	Serious ^b^	Serious ^c^	Not serious ^d^	Serious ^e^	Publication bias suspected ^f^	897 ^g^	883 ^g^	SMD: −0.58 (−1.01; −0.15)	⨁◯◯◯ Very low	Important

**Notes:** Certainty concerns the pooled anxiety outcome. ^a^ Most reports described randomized trials; Shehab et al. [21] reported uncertain randomization. ^b^ Unclear allocation, unblinded self-report, missing data and mixed analysis populations. ^c^ I^2^ = 85.0%; 95% prediction interval −2.38 to 1.22. ^d^ No additional downgrade for the broad review question; varied exercise and relaxation interventions limit modality-specific applicability. ^e^ The confidence interval spans small to large, standardized effects; clinical importance on individual scales is uncertain. ^f^ Egger’s *p* = 0.022. Trim-and-fill imputed 0 studies. ^g^ N = 1,780 represents the selected analysis populations, with shared controls counted once; adjusted-model counts are 110/112 for Rogers and 93/89 for Travier. CI, confidence interval; SMD, standardized mean difference. ⊕◯◯◯, very low certainty.

**Table 3:** Characteristics of included studies.

Study	Treatment Stage	Diagnosis Stage	Sample Size	Mean Age	Supervised or Home- Based	Frequency (per Week)	Each Session (Minutes)	Intervention	Duration	Measure Instrument	Main Outcomes
Barcelos de Souza et al., (2025) [40]	Adjuvant HT/post-treatment	Stage I–III	n = 21 E = 10 C = 11 (final)	58.70	Supervised	2 days	60	Dance exercise Free dance followed by dance therapy in the same group, separated by a 35-day washout; warm-up, movement and relaxation	12 + 12	BAI	Anxiety improvement was reported after dance therapy within a sequential program. Narrative synthesis: sequential interventions and non-concurrent assessments
Cadmus et al. (2009) [25] IMPACT	During treatment	Stage 0–IIIA	n = 50 E = 25 C = 25	54.25	Home-Based	5 days	30	Aerobic exercise Moderate-to-vigorous aerobic exercise (60–80% HR_max_; walking-based)	26	STAI state	Selected endpoint: SMD −0.13 (95% CI −0.68 to 0.43)
Cadmus et al. (2009) [25] YES	Post-treatment	Stage 0–IIIA	n = 74 E = 37 C = 37	55.79	Mixed	5 days	30	Aerobic exercise Independent YES trial; aerobic exercise progressing to 150 min/week, with supervised and home sessions	26	STAI state	Selected endpoint: SMD −0.14 (95% CI −0.60 to 0.31)
Cantarero-Villanueva et al. (2012) [43]	Post-treatment	Stage I–IIIA	n = 67 E = 32 C = 35	48.48	Supervised	3 sessions	90	Multimodal rehabilitation Core stability exercises and myofascial release massage, with progressive exercise and recovery components	8	POMS tension/anxiety	Selected endpoint: SMD −1.11 (95% CI −1.63 to −0.59)
Charati et al. (2022) [44]	Post-surgery	Non- meta- static	n = 70 E = 35 C = 35	40.39	Home-based	Walking: 3–5 days; twice daily	Walking: 10–30; stretching time NR	Combined exercise Progressive walking and shoulder stretching; walking increases from 10 to 30 min, twice daily	5	HADS-A	Selected endpoint: SMD −1.30 (95% CI −1.81 to −0.78)
Cramer et al. (2015) [45]	Post-treatment	Stage I–III	n = 40 E = 19 C = 21	49.20	Mixed	1 class + daily home practice	Class: 90; home: NR	Yoga and meditation Hatha yoga and meditation, with a weekly supervised class and recommended daily home practice	12	HADS-A	Selected endpoint: SMD 0.11 (95% CI −0.51 to 0.73)
Jose and Cacodcar (2025) [19]	Post-surgery	Stage I–IIIB	n = 58 E = 29 C = 29	57.78	Mixed	Daily; twice daily home practice	15–20 at home	Relaxation exercise Progressive muscle relaxation, deep breathing and guided imagery, with home practice; preprint report	2	SAS	Selected endpoint: SMD −1.97 (95% CI −2.61 to −1.34)
Drozd et al. (2026) [42]	During CT	Stage I–III	n = 20 E = 10 C = 10 (completers)	E: 44.10 C: 49.40	Supervised	3 sessions	45	Aerobic exercise Supervised intermittent aerobic training on a cycle ergometer; intensity individually adjusted to target heart rate	12	HADS-A	Anxiety increased from 6.9 to 9.8 in E and 7.9 to 8.7 in C. Narrative synthesis: anxiety-specific respondent counts were unclear
Gokal et al. (2016) [46]	During CT	Stage I–III	n = 50 E = 25 C = 25	52.22	Home-Based	5 days	Target 30	Home-based walking Progressive moderate-intensity walking, targeting 150 min/week	12	HADS-A	Selected endpoint: SMD −0.63 (95% CI −1.20 to −0.06)
Ho et al. (2016) [47]	During RT	Stage 0–III	n = 130 E = 66 C = 64	48.90	Supervised	2 days	90	Dance exercise Stretching, relaxation, movement games and rhythmic upper-body movement	3	HADS-A	Selected endpoint: SMD 0.17 (95% CI −0.17 to 0.52)
Kim et al. (2020) [48]	Post-treatment	Stage I–III	n = 48 E = 23 C = 25	49.17	Mixed	1 day for 6 weeks; 2 days for 6 weeks	Exercise duration NR	Combined exercise Aerobic and resistance exercise with social support; the 2-h social meetings are distinct from exercise sessions	12	HADS-A	Selected endpoint: SMD 0.03 (95% CI −0.53 to 0.60)
Larkey et al. (2025) [41]	Post-treatment	Stage 0–III	n = 149 TCQ: 49 SQG: 45 ES: 55 (week 8)	59.87	Mixed	1 class + home practice 4–5 days	Class: 60; home: ≥30	Tai Chi/Qigong TCQ with breathing and meditative focus; sham Qigong is an active comparator; education support is the third arm	8	POMS anxiety	No clear overall anxiety benefit; TCQ benefit was reported in the moderately fatigued subgroup. Narrative synthesis: adjusted model estimates, without usable raw SDs
Lavín-Pérez et al. (2025) [26]	Post-treatment	Early breast cancer	n = 54 E = 36 C = 18 (combined)	50.30	Supervised online	3 days	60–70	Multicomponent training HIIT and resistance exercise; HRV-guided and preplanned exercise arms combined against one control group	16	HADS-A	Selected endpoint: SMD −0.65 (95% CI −1.23 to −0.07)
Liu et al. (2022) [49]	During Adjuvant CT	Stage I and II	n = 124 E = 61 C = 63	NR	Supervised	1 day	90	Mindfulness yoga 20 min meditation, 50 min yoga with breathing, 10 min body scan and 10 min sensory summary	8	HADS-A	Selected endpoint: SMD −0.42 (95% CI −0.77 to −0.06)
Mehnert et al. (2011) [50]	Post-treatment	Stage I–III	n = 58 E = 30 C = 28	51.88	Supervised	2 days	90	Physical exercise rehabilitation Indoor movement-based activities and outdoor aerobic training	10	HADS-A	Selected endpoint: SMD −0.54 (95% CI −1.06 to −0.01)
Moraes et al. (2021) [51]	Post-treatment	Unspeci- fied	n = 25 E = 12 C = 13	54.60	Supervised	1 day	NR	Resistance exercise Supervised machine- and free-weight resistance training	8	STAI state	Selected endpoint: SMD −0.08 (95% CI −0.86 to 0.71)
Ozkan et al. (2022) [52]	During RT	Stage I–III	n = 20 E = 10 C = 10	45.50	Supervised	3 days	Aerobic: 40; resistance: NR	Combined exercise Aerobic treadmill training followed by resistance exercise	6	BAI	Selected endpoint: SMD −0.19 (95% CI −1.07 to 0.69)
Rao et al. (2008) [53]	Pre/Post-surgery	Stage II–III	n = 69 E = 33 C = 36	49.20	Mixed	NR	NR	Integrated yoga Pranayama breathing and yogic relaxation around surgery, with home practice	4	STAI state	Selected endpoint: SMD −0.68 (95% CI −1.16 to −0.19)
Rogers et al. (2017) [28]	Post-treatment	Stage 0–IIIA	n = 222 E = 110 C = 112 (model)	54.40	Mixed	Full schedule NR	NR	Aerobic exercise Supervised-to-home exercise with behavioral support; target approximately 150 min/week	12	HADS-A	Adjusted selected endpoint: SMD −0.33 (95% CI −0.53 to −0.14)
Shehab et al. (2022) [21]	During Adjuvant CT/RT	Unspeci- fied	n = 48 E = 24 C = 24	47.12	Supervised	2 sessions	30	Aerobic exercise Upper- and lower-body ergometer exercise with progressive intensity; randomization unclear	4	HADS-A	Selected endpoint: SMD −4.96 (95% CI −6.14 to −3.78)
Travier et al. (2015) [27]	During Adjuvant CT/RT	Non- meta- static (M0)	n = 182 E = 93 C = 89 (model)	49.60	Mixed	2 classes + 3 home days	Class: 60; home: ≥30	Combined exercise Supervised aerobic interval and resistance training, plus exercise at home on three other days	18	HADS-A	Adjusted selected endpoint: SMD 0.06 (95% CI −0.19 to 0.31)
Wei et al. (2022) [54]	During CT	Stage I–III	n = 67 E = 32 C = 35	NR	Mixed	5 days	30	Baduanjin exercise Initial hospital instruction followed by video-guided home exercise, including warm-up and relaxation	12	HADS-A	Selected endpoint: SMD −0.46 (95% CI −0.95 to 0.02)
Ying et al. (2019) [29]	Post-treatment	Unspeci- fied	n = 86 E = 46 C = 40	54.09	Mixed	3 classes + 4 home days	Class: 60; home: ≥20	Baduanjin exercise Three supervised classes plus home practice on four other days each week	26	GAD-7	Selected endpoint (t-derived): SMD 0.03 (95% CI −0.39 to 0.46)
Zhang et al. (2022) [55]	Post-treatment	Stage I–III	n = 58 E = 29 C = 29	47.50	Supervised	2 days	60	Mindfulness Tai Chi Chuan 24-form Tai Chi with breathing and mindfulness focus; 60-min complete sessions	8	SAS	Selected endpoint: SMD −0.67 (95% CI −1.20 to −0.14)
Zhang et al. (2023) [20]	During Adjuvant CT/RT	Stage I–IV	n = 180 E = 90 C = 90	48.93	Supervised	1 class + 6 practice days	60	Multimodal exercise Aerobic, resistance, flexibility and acupoint components; weekly teaching and supervised practice in the ward	5	HADS-A	Selected endpoint: SMD −0.24 (95% CI −0.53 to 0.06)

Notes: E, exercise; C, control; CT, chemotherapy; RT, radiotherapy; HT, hormone therapy; NR, not reported or not sufficiently specified; HR_max_, maximum heart rate; HRV, heart rate variability; HIIT, high-intensity interval training; TCQ, Tai Chi/Qigong; SQG, sham Qigong; ES, education support. BAI, Beck Anxiety Inventory; STAI, State-Trait Anxiety Inventory; POMS, Profile of Mood States; HADS-A, Hospital Anxiety and Depression Scale anxiety subscale; SAS, Self-Rating Anxiety Scale; GAD-7, Generalized Anxiety Disorder-7; SMD, standardized mean difference; CI, confidence interval; SD, standard deviation.

**Table 4:** Heterogeneity statistics.

Q	Degrees of Freedom	*p* value	I^2^ (%)	τ^2^
139.90	21	<0.0001	85.0	0.7001

**Table 5:** Egger’s regression test for funnel plot asymmetry.

Intercept	Standard Error	t	Degrees of Freedom	*p* Value
−3.052	1.233	−2.48	20	0.022

**Table 6:** Meta-regression analyses for continuous moderators.

Moderator	k	Estimate (β)	SE	t	*p*	95% CI	R^2^ (%)
Baseline HADS-A (intervention group; points)	13	−0.2551	0.0901	−2.829	0.0164	[−0.4535, −0.0566]	46.45
Mean age (years)	20	0.0453	0.0606	0.747	0.4649	[−0.0821, 0.1727]	0.00
Program duration (weeks)	22	0.0504	0.0269	1.876	0.0753	[−0.0056, 0.1065]	17.18
Frequency (days/week)	19	0.0571	0.1188	0.481	0.6369	[−0.1936, 0.3079]	0.00
Total sample size (n)	22	0.0039	0.0039	0.986	0.3359	[−0.0043, 0.0120]	0.00
Session duration (minutes)	13	0.0075	0.0137	0.546	0.5957	[−0.0226, 0.0376]	0.00
Cumulative prescribed dose (minutes)	13	0.000275	0.000183	1.505	0.1606	[−0.000127, 0.000677]	11.19

k, independent trial estimates with available moderator data; β, change in Hedges’ g per one-unit increase in the stated moderator; SE, standard error; CI, confidence interval. Negative β indicates a larger reduction in anxiety as the moderator increases. Each row is a separate model; *t* tests use k − 2 degrees of freedom. *p* values are unadjusted; none meets the seven-test threshold of 0.0071. R^2^ is estimated on the same subset as its corresponding model. Baseline anxiety includes HADS-A studies only.

## Data Availability

No new data were generated; all used data were already published.
